# Sourcing and framing in cancer control continuum: A content analysis of Malaysian English and Chinese online cancer news

**DOI:** 10.3389/fpubh.2022.924027

**Published:** 2022-12-01

**Authors:** Thomas Hongjie Zhang, Jen Sern Tham, Xueyong Yu, Peng Kee Chang, Suet Nie Kho

**Affiliations:** ^1^Department of Communication, Faculty of Modern Languages and Communication, Universiti Putra Malaysia, Seri Kembangan, Malaysia; ^2^School of Communication, Taylor's University, Subang Jaya, Malaysia; ^3^Centre for Research in Media and Communication, Universiti Kebangsaan Malaysia, Bangi, Malaysia

**Keywords:** sourcing, framing, cancer control continuum, cancer risk communication, health promotion, ethnicity and health, quantitative content analysis

## Abstract

**Background:**

In health news production, sourcing and framing are two critical mechanisms that influence how newsreaders think about and perceive the severity of a health issue. Understanding how local media covers the cancer control continuum is vital. However, very limited studies have looked at the effect of sourcing and framing in cancer news coverage, and it is still unknown how sources and news frames shape cancer coverage, especially in non-Western countries.

**Objective:**

This study examines framing and sourcing patterns in news stories reporting on cancer control in Malaysian mainstream (English) and ethnicity (Chinese) online news sites, uncovering underlining associations between essential news components, source, and framing.

**Methods:**

We used a predesigned code book to conduct a quantitative content analysis on cancer news stories (*n* = 841) published on two Malaysian English and Chinese online news sites from 2017 to 2019. Cancer news received adequate coverage in Malaysian English and Chinese media and was also session-centered.

**Results:**

Two logistic regression models demonstrated the internal relationships between sourcing, framing, and different elements in cancer coverage. In terms of news sources, the results revealed that medical journals were the most likely to be cited when the news focused on medical research, followed by primary cancer prevention. When the news concentrated on statistical cancer reports and environmental/occupational risk factors, government agencies were more likely to be interviewed. Of news frames, when the news articles engaged with medical institutions and mentioned medical publications, the lifestyle frame was very likely to be shown, but the environmental frame was more likely to be portrayed when interviewing medical practitioners.

**Conclusion:**

This study is the first comprehensive assessment to analyze and compare Malaysian English and Chinese online cancer news coverages and uncover underlying associations between news components, sourcing, and framing paradigms. We contributed to the scholarly understanding of cancer news coverage. This study can serve as a model for future health promotion researchers, journalists, and policymakers. Implications for cancer risk communication research, health journalist practices, and health policymaking were discussed.

## Introduction

Cancer is one of the dominant non-communicable diseases. It is a crucial and long-term national health problem in Malaysia. The top three types of cancer suffered by Malaysians are breast cancer (17.3%), colon cancer (14.0%), and lung cancer (10.7%) (1). As a multi-ethnicity country, there is a sizeable racial gap in cancer incidence rates. According to the Malaysian National Cancer Registry Report (2012–2016), cancer is most commonly detected among Chinese Malaysians when compared with the other two major ethnicities, i.e., Malay and Indian (1). Scientific evidence shows that approximately half of all cancer cases reported today can be prevented if the public practice a healthy lifestyle and avoids risky behavior that may develop cancer (2). Therefore, increasing awareness and cancer literacy among the public in Malaysia is imperative to promote behavioral change. In particular, media channels play an influential role in creating public awareness about cancer (3). News media holds substantial promise as a tool for reaching and persuading people to adopt new and healthier lifestyles (4). Health news coverage could be treated as a “translated script” for medical issues, which converts “medical language” or other obscure jargon to “public language” (5). Nowadays, newsreaders can intentionally and unintentionally engage with information about the cancer control continuum during daily media engagements. Their awareness, knowledge level, and preventive intention on cancer issues would be increased during cancer information engagement (6).

The nature of cancer information presentation in the media can be understood in two classic journalistic viewpoints, framing, and sourcing. First, journalists usually select, emphasize, and highlight specific health issues based on pre-established frames to encourage readers to think and discuss the issues in particular framed ways (7, 8). Up to date, framing research has gained much scholarly attention in public health communication domains (9). Framing is a concept that explains how the media portray health news and how the different portrayals impact health-related behaviors among the readers (10). To date, many studies scrutinized the diversified framing practices on the concerns of global cancer epidemiology. For example, Riles et al. (8) found that the majority of cancer news from mainstream American online news sites were portrayed by the medical frame, highlighting news elements based on medical facts rather than environmental or lifestyle concerns; this trend has also increased over time. Similarly, Clarke and Everest's (11) results also showed that the medical frame was portrayed in most cancer news articles from American and Canadian magazines, followed by the lifestyle frame. Meanwhile, there are other dimensions to understand the frame portrayals in cancer news. In the context of South Korea, researchers revealed that more than half of cancer news coverage written by professional health journalists was rooted in the personal (epidemic) frame instead of the social (thematic) frame (12). This phenomenon was also observed in cancer news in English on Facebook; most of the news on different types of cancer was presented *via* the epidemic frame (13). Of note, different news frames can affect news readers' thinking about cancer, such as the perception of cancer issues, the attitude toward cancer survivors (8), and the level of cancer awareness and screening/treatment intentions (14).

Furthermore, apart from framing, another critical paradigm for understanding cancer news coverage is the invitation of news sources or sourcing approaches. Different news sources would provide helpful information or suggestions from various backgrounds, shape news content in specific ways, and influence how the issues would be presented (15), particularly when it comes to public health concerns (16). Therefore, inviting news sources is a pivotal element in the frame-building process (17). Research using content analysis methods on cancer coverage has found that elite parties such as physicians and government officers are the predominant information sources to which health journalists usually refer. For instance, one study examining cancer coverage in the USA reported that national medical institutions, including the National Cancer Institute (NCI) and medical schools at research universities, were frequently cited in cancer news (18). Recently, Peng et al. (19) also found that medical institutions, healthcare providers, and scientists were the major sources providing evidence regarding the causality of cancer risk factors in cancer news from American newspapers. Besides, regarding news coverage on cervical cancer and HPV, physicians, government agencies, and medical institution such as the Centers for Disease Control and Prevention (CDC) were once again the primary sources providing helpful information to the journalists (20). These findings demonstrate a general picture of what sources are preferred in cancer news across contexts and timeframes.

Although existing studies have analyzed news framing and sourcing techniques in cancer news coverage, several gaps remain and are waiting to be addressed. First, most studies focused on cancer news from the Western context, especially news coverage from American media channels (8, 18). It remains unclear how the Eastern and non-English media practice framing and sourcing paradigms. Second, the foci and involvements of frame type are inconsistent among studies that examined news frames in cancer coverages. Some studies employed the responsibility-blaming frame (i.e., episodic frame and thematic frame) (13, 21, 22), while others conceptualized the issue-centered frame for cancer news (i.e., medical frame, environmental frame, and lifestyle frame) and then analyzed it descriptively (8, 11). Even though the issue-centered frame generally portrays the presentation patterns of the cancer control continuum, previous findings can only present the phenomena during a given timeframe and in a specific context; cross-time and context framing analyses for cancer news are needed. Third, for sourcing paradigms, most published studies only touched on percentages of source involvement and then generalized that medical sources are the most popular in cancer news (18, 23); in-depth analyses for demonstrating the associations between source invitations, presentation of cancer control continuum, frame portrayals, and other news factors are still lacking.

Taken all together, this study intends to analyze framing and sourcing practices in cancer news in Malaysian news sites, an eastern research context. The media environment in Malaysia is highly racial-centered or ethnicity-divided as the population comprises three major ethnic groups, namely, Malay, Chinese, and Indian (24). As the Chinese have the highest cancer incidence rate (25), it is reasonable for us to study how the cancer control continuum is presented in the media and educate Chinese community members about cancer issues. While sourcing and framing practices are two vital components in health news reporting, it is thus essential to unveil its underlying mechanisms in cancer news published by Malaysian media. Therefore, we seek to analyze framing and sourcing practices in cancer news from Malaysian Chinese ethnicity media and mainstream English media and uncover internal associations between essential news components, source, and framing.

## Methods

Quantitative content analysis was carried out for this study. Malaysian English and Chinese online news sites were the units for coding. The news sites need to meet the following requirements: (1) News articles on the website are published in English or Chinese; (2) the websites are owned by major local newspapers; (3) the newspapers are daily newspapers that could be purchased in Peninsular Malaysia, Sarawak, and Sabah; (4) the news websites are public accessible without subscription payment; and (5) the newspaper websites occupied the highest usage among several other Malaysian news websites from the same language category. We referred to the Reuters Institute Digital News Report 2020 (26) and found that *The Star Online* (belongs to *The Star*) and *Sin Chew Online/*星洲网 (belongs to *Sin Chew Daily*/星洲日报) met the requirements mentioned above. We treated each headlined cancer news article published in *The Star Online* and *Sin Chew Online* as the unit of analysis for analytical purposes.

As the sampling process was conducted in February 2020, there still did not have an intact and most recent news content for the entire year 2020. After considering the workload and the level of representability, we decided to select cancer news articles published in a three-year duration from January 2017 to December 2019, which are the closest to 2020. Furthermore, the manual coding was guided by particular coding keywords, such as cancer, cancer prevention, cancer screening, lung cancer, and breast cancer. Based on the keywords, an independent news article would be selected if any sampling keyword appeared in its headline, sub-headline, or the first three paragraphs (including the lead). Based on the keywords searching on the two news sites, 841 cancer news articles were identified and included in the data analysis (*N* = 841), including 436 from The Star Online (51.8%) and 405 from Sin Chew Online (48.2%).

Four coding items were developed based on previous studies to scrutinize each defined unit of analysis; the detailed conceptualization for each coding item is attached in Supplementary material. Two English and Chinese bilingual coders were trained and collaborated on the coding work. Two coders agreed to discuss and seek solutions if any problem occurred during the coding process. We conducted an inter-coder reliability test with a smaller sample from the complete data to assess the coding consistency. According to Riffe et al.'s (27) formula for calculating sample size for the inter-coder reliability test, the two coders double-coded 10.7% (*n* = 90) of the sample. Cohen's kappa values fall between 0.71 and 1.0, demonstrating sufficient agreements (28).

Two statistical methods in SPSS version 26 were applied to analyze the news data, descriptive analysis, and logistic regression. The analysis methods were carried out based on the research objectives. The descriptive analysis explored the patterns and news factors in online cancer news. Logistic regression was applied to uncover the associations between news factors and the portrayal of news frames, as well as the invitation of news sources.

## Results

### General findings

**Figure 1:**
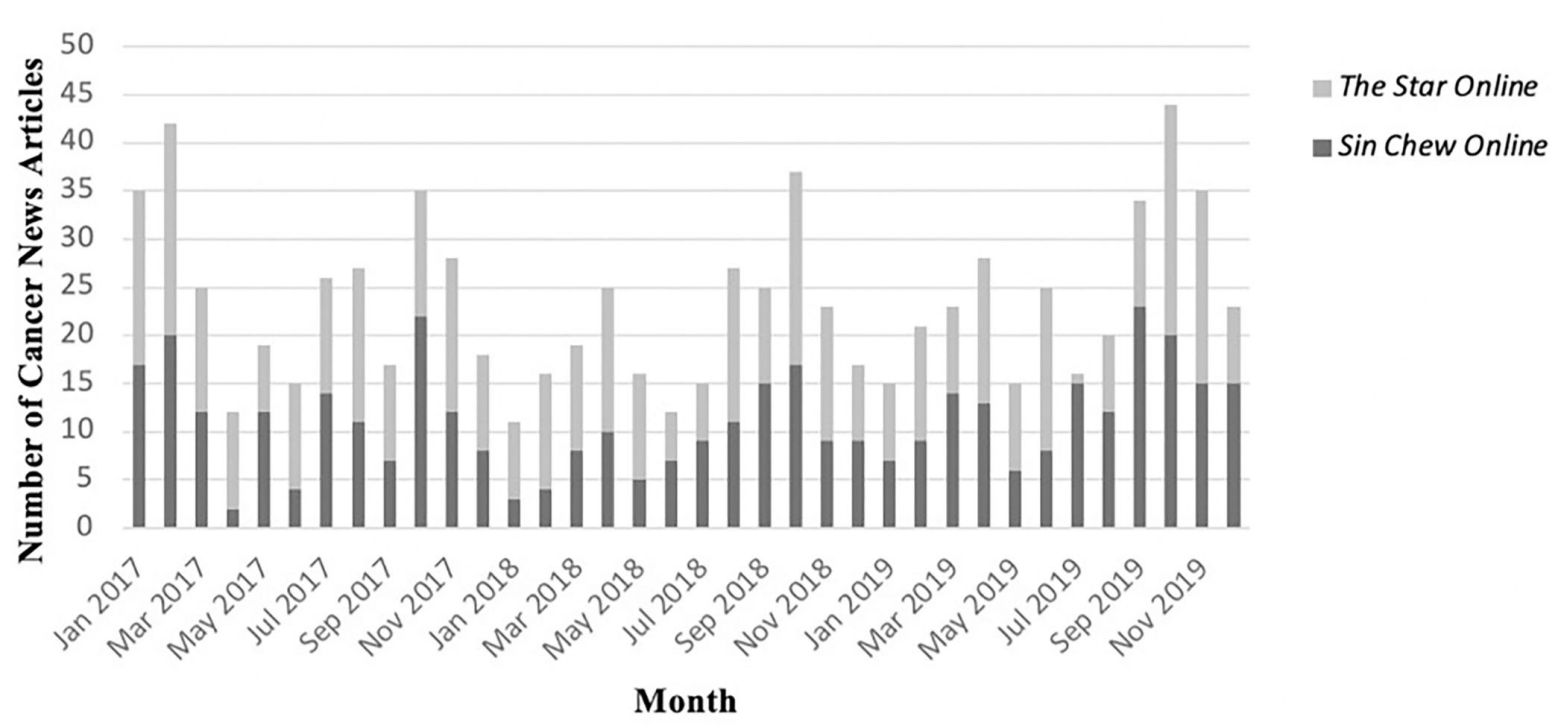
Number of cancer news articles reported by The Star Online and Sin Chew Online (*N* = 841).

Figure 1 shows the monthly distribution of cancer news articles for The Star Online and Sin Chew Online from January 2017 to December 2019 (*N* = 841). Both news sites reported 10–15 cancer news articles in the majority of the months. A finding of interest was that both news sites covered more than 20 cancer news articles on cancer-related information in certain months, including the peaks shown in Figure 1. Seven peaks in the figure indicated that more than 30 cancer news stories were reported in the respective months of January 2017, February 2017, October 2018, September 2019, October 2019, and November 2019. In February 2017 and October 2019, more than 40 news articles focused on cancer issues from the two news sites. As expected, the Chi-square test indicated no significant difference in the number of cancer news articles covered by The Star Online and Sin Chew Online during the 3 years [*x*^2^ = (564, *N* = 841) =583.98, *p* = 0.272].

### Predicting the invitation of news sources

Binary logistic regression was employed to understand the associations between different news components and the invitation of each news source (Table 1). We treated the cancer control continuum and cancer risk factors as predictors. As all factors were dichotomous, we built logistic regression models to examine the relationships between news components and sourcing practices. First, the regression results showed that medical journals were more likely to be referenced when the news article focused on medical research (OR: 6.43, 95% CI: 2.85–14.54, *p* < 0.001), primary cancer prevention (OR: 2.80, 95% CI: 1.28–6.12, *p* = 0.010), and mentioned lifestyle risks (OR: 5.96, 95% CI: 3.09–11.48, *p* < 0.001), but less likely to be referenced when the news covered medical treatment (OR: 0.18, 95% CI: 0.04–0.78, *p* = 0.023). Second, our results also revealed that experts or staff from medical institutions were more likely to be interviewed when the news article embarked on medical research (OR: 11.86, 95% CI: 6.52–21.56, *p* < 0.001), secondary cancer prevention (OR: 2.50, 95% CI: 1.74–3.59, *p* < 0.001), medical treatment (OR: 1.65, 95% CI: 1.10–2.46, *p* = 0.015), as well as mentioned lifestyle risks (OR: 2.40, 95% CI: 1.74–3.33, *p* < 0.001) and medical risks (OR: 1.98, 95% CI: 1.48–2.64, *p* < 0.001), but less likely to be interviewed when the cancer news focused on social support/survivorship topics (OR: 0.26, 95% CI: 0.17–0.38, *p* < 0.001). Third, pharmaceutical companies were less likely to be interviewed when the cancer news focused on primary prevention (OR: 0.22, 95% CI: 0.06–0.97, *p* = 0.031).

**Table 1:** Binary logistic regression analysis: associations between news factors and the invitation of news sources (*N* = 841).

**Predictors**	**News sources Odds ratio [95% CI]**				
	**Medical Journal**	**Medical Institution**	**Pharmaceutical Company**	**Government Agency**	**NGOs**
**Cancer continuum**					
Primary cancer prevention (ref: No)	2.80^**^ [1.28–6.12]	1.34 [0.92–1.93]	0.22^*^ [0.06–0.87]	0.74 [0.49–1.13]	1.59^*^ [1.10–2.31]
Secondary cancer prevention (ref: No)	0.04 [0.16–1.01]	2.50^***^ [1.74–3.59]	1.87 [0.52–6.73]	0.73 [0.48–1.13]	0.73 [0.50–1.08]
Medical treatment (ref: No)	0.18^*^ [0.04–0.78]	1.65^**^ [1.10–2.46]	1.34 [0.40–4.49]	0.85 [0.54–1.34]	0.30^***^ [0.19–0.47]
Social support/survivorship (ref: No)	/	0.26^***^ [0.17–0.38]	/	0.80 [0.50–1.26]	3.18 [2.13–4.75]
Medical research (ref: No)	6.43^***^ [2.85–14.54]	11.86^***^ [6.52–21.56]	0.67 [0.15–3.03]	0.22^***^ [0.11–0.44]	0.23^***^ [0.12–0.41]
Statistical report (ref: No)	1.13 [0.40–3.24]	0.75 [0.46–1.23]	1.02 [0.21–4.92]	3.55^***^ [2.18–5.76]	0.66 [0.3−1.15]
**Cancer risk factors**					
Lifestyle risks (ref: No)	5.96^***^ [3.09–11.48]	2.40^***^ [1.74–3.33]	0.44 [0.10–2.03]	0.60^*^ [0.39–0.92]	0.56^**^ [0.39–0.79]
Environmental/occupational risks (ref: No)	0.48 [0.06–3.71]	0.67 [0.29–1.57]	/	2.88^*^ [1.21–6.85]	1.12 [0.46–2.72]
Demographical risks (ref: No)	0.57 [0.30–1.10]	0.90 [0.68–1.19]	0.58 [0.19–1.81]	0.89 [0.48–1.02]	1.53^**^ [1.13–2.07]
Medical risks (ref: No)	0.60 [0.30–1.22]	1.98^***^ [1.48–2.64]	0.81 [0.26–2.52]	0.70 [0.48–1.02]	0.56^***^ [0.30–1.19]

The

*, **, and *** symbols indicates the values of *p* < 0.05, *p* < 0.01, and *p* < 0.001 respectively.

In terms of government agencies, it was more likely to be interviewed when the news focused on cancer statistical report (OR: 3.55, 95% CI: 2.18–5.76, *p* < 0.001) and mentioned environmental/occupational risk factors (OR: 2.88, 95% CI: 1.21–6.85, *p* = 0.017); however, it was less likely to be interviewed when the news focused on medical research (OR: 0.22, 95% CI: 0.11–0.44, *p* < 0.001) and mentioned lifestyle risks (OR: 0.60, 95% CI: 0.39–0.92, *p* = 0.019). When it comes to NGOs, they were more likely to be interviewed when the news focused on primary cancer prevention (OR: 1.59, 95% CI: 0.39–0.92, *p* = 0.019) and mentioning demographical risks (OR: 1.53, 95% CI: 1.13–2.07, *p* = 0.006), but less likely to be interviewed when the news focused on medical treatment (OR: 0.30, 95% CI: 0.19–0.47, *p* < 0.001), medical research (OR: 0.23, 95% CI: 0.12–0.41, *p* < 0.001), as well as mentioned medical risks (OR: 0.56, 95% CI: 0.30–1.19, *p* < 0.001) and lifestyle risks (OR: 0.56, 95% CI: 0.39–0.79, *p* = 0.001).

### Predicting the portrayal of news frames

The relationships between news components and the portrayal of each news frame in cancer news from The Star Online and Sin Chew Online were examined (Table 2). There are three types of issue-centered news frames applied in this study; due to the nature of measurement and each news article being required to determine only one primary news frame, we conducted multinominal logistic regression. First, the lifestyle frame was more likely to be portrayed when the news article engaged with medical institutions (OR: 7.63, 95% CI: 3.82–15.25, *p* < 0.001), citing medical journals (OR: 17.46, 95% CI: 2.23–137.00, *p* = 0.007), focusing topics on primary cancer prevention (OR: 96.83, 95% CI: 34.75–269.82, *p* < 0.001), medical research (OR: 8.99, 95% CI: 3.12–25.90, *p* < 0.001), as well as mentioned lifestyle risks (OR: 11.14, 95% CI: 6.17–20.12, *p* < 0.001). However, the lifestyle frame was absent when the cancer news focused on social support/survivorship-related topics (OR: 0.10, 95% CI: 0.04–0.22, *p* < 0.001). Besides, the environmental frame was more likely to be portrayed in the cancer news when interviewing staff from medical institutions (OR: 7.25, 95% CI: 1.78–29.57, *p* = 0.006), but less likely to be portrayed when the news focused on social support/survivorship (OR: 0.09, 95% CI: 0.02–0.48, *p* = 0.005).

**Table 2:** Multinominal logistic regression analysis: associations between news factors and the portrayal of news frames (*N* = 841).

**Predictors**	**News frames Odds ratio [95% CI] (Reference: no clear identified)**		
	**Lifestyle frame**	**Environmental frame**	**Medical frame**
**News source**			
Medical journal	17.46^**^ [2.23–137.00]	/	2.56 [0.33–19.94]
Medical institution	7.63^***^ [3.82–15.25]	7.25^**^ [1.78–29.57]	5.31^***^ [3.10–9.09]
Pharmaceutical company	/	/	3.42 [0.42–27.72]
Government agency	0.81 [0.39–1.71]	2.83 [0.73–10.95]	0.75 [0.45–1.24]
NGOs	1.13 [0.60–2.12]	1.05 [0.27–4.07]	0.79 [0.50–1.26]
**Cancer control continuum**			
Primary cancer prevention	96.83^***^ [34.75–269.82]	4.37 [0.96–19.93]	3.91^***^ [2.23–6.88]
Secondary cancer prevention	1.33 [0.42–4.21]	0.91 [0.08–9.74]	17.73^***^ [6.70–46.69]
Medical treatment	0.99 [0.37–2.69]	/	2.65^***^ [1.52–4.65]
Social support/survivorship	0.10^***^ [0.04–0.22]	0.09^**^ [0.02–0.48]	0.34^***^ [0.19–0.61]
Medical research	8.99^***^ [3.12–25.90]	3.72 [0.59–23.26]	5.64^***^ [2.32–13.70]
Statistical report	1.03 [0.32–3.30]	/	1.47 [0.61–3.54]
**Cancer risk factors**			
Lifestyle risks	11.14^***^ [6.17–20.12]	1.06 [0.14–8.01]	1.80^*^ [1.06–3.07]
Environmental/occupational risks	/	/	/
Demographical risks	1.08 [0.64–1.80]	0.82 [0.19–3.65]	2.04^***^ [1.38–3.04]
Medical risks	1.44 [0.82–2.53]	2.06 [0.48–8.92]	3.10^***^ [2.02–4.76]

* p < 0.05.

** p < 0.01.

*** p < 0.001.

As most of the cancer news articles portrayed the medical frame (*n* = 576, 68.5%), the regression results showed many news components significantly associated with the portrayal of the medical frame. It was more likely to be portrayed when the cancer news interviewed staff from medical institutions (OR: 5.31, 95% CI: 3.10–9.09, *p* < 0.001), focused on topics regarding secondary cancer prevention (OR: 17.73, 95% CI: 6.70–46.69, *p* < 0.001), followed by medical research (OR: 5.64, 95% CI: 2.32–13.70, *p* < 0.001), primary cancer prevention (OR: 3.91, 95% CI: 2.23–6.88, *p* < 0.001), medical treatment (OR: 2.65, 95% CI: 1.52–4.65, *p* < 0.001), as well as mentioned medical risks (OR: 3.10, 95% CI: 2.02–4.76), demographical risks (OR: 2.04, 95% CI: 1.38–3.04, *p* < 0.001), and also lifestyle risks (OR: 1.80, 95% CI: 1.06–3.07, *p* = 0.031).

## Discussion

This study first presented the patterns of cancer coverages from Malaysian two dominant English and Chinese online news sites, namely, *The Star Online* and *Sin Chew Online*, for a 3-year duration (2017–2019) and subsequently uncovered the associations between news components, framing, and sourcing practices. First, regarding the patterns of Malaysian cancer news coverages, we found a relatively consistent monthly distribution from January 2017 to December 2019. There were usually 15–20 cancer news articles reported by *The Star Online* and *Sin Chew Online* each month. From this, we can notice that Malaysian news media have already considered cancer-related coverage an essential part of their routine health news production. The local media, especially online news platforms, act as the “top-down mechanism” health agenda setters for global cancer issues (29). Meanwhile, it is worth and essential to look at the peaks and nadirs. We found more than 30 cancer news articles covered by the two news sites in certain months, but in some months, there were <15 articles that focused on the cancer control continuum. In January, February, September, October, and November, the total number of cancer news articles was more than 30. The possible reasons would be that World Cancer Day falls in February every year, the Children Cancer Awareness Month, Breast Cancer Awareness Month, and Lung Cancer Awareness Month are located in each September, October, and November. Thus, there would be more cancer awareness events, campaigns, or workshops in these months than at other times in the year. It is slightly consistent with empirical findings from other countries; cancer awareness months gain much more journalistic attention, and appropriate coverages always mushroomed during these months (30, 31). Oppositely, during May, June, and July, the number of cancer news articles was somehow dropped, subject to Malaysians from different ethnic groups celebrating several festivals during these 3 months, such as Eid al-Fitr, King's Birthday, Wesak Day, Gawai Dayak, and Dragon Boat Festival. The news media tend to focus more on positive events than disease-relevant stories. Thus, we can understand that Malaysian cancer news coverage is generally session-centered (30).

Next, two regression models were built to predict the associations between news components and sourcing and framing paradigms. For the invitation of news sources, we ran binary logistic regression for each type of source accordingly. As the elite source in the health news coverage (16), experts from medical institutions were the prominent news source in our sample. Our results showed that when the news focused on medical research (OR: 11.86, 95% CI: 6.52–21.56, *p* < 0.001) and mentioned lifestyle risks (OR: 2.40, 95% CI: 1.74–3.33, *p* < 0.001), medical institutions were most likely to be invited. Medical institutions were also likely to be interviewed when the news looked at secondary cancer prevention (e.g., screening and detection) (OR: 2.50, 95% CI: 1.74–3.59, *p* < 0.001), medical treatment (OR: 1.65, 95% CI: 1.10–2.46, *p* = 0.015), and mentioning medical risks (OR: 1.98, 95% CI: 1.48–2.64, *p* < 0.001).

Still, the likelihood was significantly lower than those mentioned above. However, as another elite medical source, medical journals received relatively more minor attention in Malaysian cancer news, which only was cited in the news about medical research (OR: 6.43, 95% CI: 2.85–14.54, *p* < 0.001), primary cancer education (e.g., health education and promotion; OR: 2.80, 95% CI: 1.28–6.12, *p* = 0.010), and mentioned lifestyle risks (OR: 5.96, 95% CI: 3.09–11.48, *p* < 0.001); the odds ratios were lower than medical institutions. In the same vein as Moriarty et al.'s (18) finding, our results indicated that Malaysian news sites rarely engage with pharmaceutical companies in cancer news coverage. Once again, this phenomenon confirmed that the cancer control continuum is a non-commercial-oriented medical agenda; medical practitioners and the government play the central role in coping with it (16, 23).

Different from previous sourcing studies in health promotion (23, 24), our findings indicated that non-medical elite sources are somewhat equally important, such as government agencies and NGOs. For government agencies, we only found that it was more likely to be invited when the news was presenting cancer statistics (e.g., incident rates, mortality rates, and cancer type distributions; OR: 3.55, 95% CI: 2.18–5.76, *p* < 0.001) and mentioning environmental/occupational cancer risks (e.g., the associations between pollution, radiation, and occupational exposures; OR: 2.88, 95% CI: 1.21–6.85, *p* = 0.017). Even though only two predictors showed significant positive relationships with the invitation of government agencies, it is still strong enough to draw some notes down. This finding could serve as guidance for health journalists and health promotion designers when they encounter health statistics and environment-/occupation-related issues. Officers from the government are the primary source they can refer. Pertaining to NGOs, we found that it was more likely to be engaged when the news was embarking on events or campaigns about primary cancer prevention (OR: 1.59, 95% CI: 0.39–0.92, *p* = 0.019), as well as demographical cancer risks (OR: 1.53, 95% CI: 1.13–2.07, *p* = 0.006). We found that local NGOs frequently launched cancer awareness campaigns, events, or free check-ups; meanwhile, some NGOs were demographic specific, focusing on female and children's cancers, which explained our findings. We also shall highlight that NGOs were less likely to be interviewed when the news covered topics about medical treatment, medical research, mentioned lifestyle, and medical risks. It is easy to be understood based on the characteristics of cancer NGOs, which concentrate more on the health promotion level regarding prevention and awareness rather than clinical tasks. It shed light on health journalists regarding what topics they could refer to NGOs and the other topics that need to be referring clinicians and medical staff.

Furthermore, multinominal logistic regression analysis predicted the portrayals of each type of news frame in cancer news coverages from *The Star Online* and *Sin Chew Online*. Our results reported a similar phenomenon in previous studies (8, 11). Most cancer news from the two news sites was portrayed in the medical frame (68.5%). It is not surprising that regardless of the context of the study, either from the USA or an Asian country, the nature and general functions of cancer coverage are similar, such as introducing knowledge on cancer issues, promoting awareness, and facilitating preventive actions. Of note, the regression results showed that, according to odds ratios, the medical frame was most likely to be portrayed when the cancer news interviewed medical institutions (OR: 5.31, 95% CI: 3.10–9.09, *p* < 0.001), focused on secondary cancer prevention (OR: 17.73, 95% CI: 6.70–46.69, *p* < 0.001) and mentioned medical risks (5.64, 95% CI: 2.32–13.70, *p* < 0.001). The lifestyle frame was most likely to be portrayed when the news cited medical journals focusing on primary cancer prevention (OR: 96.83, 95% CI: 34.75–269.82, *p* < 0.001) and mentioned lifestyle risks (OR: 11.14, 95% CI: 6.17–20.12, *p* < 0.001). We only found it was more likely to be portrayed for the environmental frame when the news interviewed medical institutions (OR: 7.25, 95% CI: 1.78–29.57, *p* = 0.006). Furthermore, we also found that other predictors indicated positive associations, but the odds ratios were lower than those mentioned above. Our findings on the portrayal of news frames can deepen our understanding of the issue-centered frame in cancer news. We presented the percentage of each type of frame and uncovered the internal associations between news frames and different news components. It broadened Riles et al.'s (8) rationale regarding framing research in cancer risk communication.

This study is not without limitations. First, we only managed to code and analyze news articles published in 3 years due to the workload; the representability is insufficient. Future research shall extend the time duration to a longer time, 10 years or at least 5 years, to obtain a higher level of representativeness. Second, we proposed this study based on the severity of cancer issues in the Malaysian Chinese community (25). We selected cancer news from Chinese ethnic and mainstream English media as the study sample. Initially, this study overgeneralized the patterns of cancer news in Malaysia. More cancer news articles are published in other languages, such as Malay and Tamil, used by the ethnic majority (i.e., the Malays) and the Indian community. Future research needs to look at Malaysian cancer news reported by Malay and Tamil media to confirm or argue our findings. Third, regarding the conceptualization of news sources, we only focused on a few elite sources applied in previous research and scrutinized them thoroughly. We failed to cover non-elite sources, which are also vital in health coverage (31, 32), and merge sources into groups, such as analyzing sources' ethnic and nationality backgrounds. Future research should divide sources into groups.

## Conclusion

As one of the first studies analyzed and compared Malaysian cancer news coverage from English and Chinese news sites and uncovered internal associations between news components, sourcing, and framing paradigms, we can further scholarly understanding of cancer news coverage. Unlike most previous content analysis studies that only analyzed the distribution or percentage of specific news elements, we considered elements in cancer news articles as predictors and explored the mechanisms behind two essential news production processes. It can guide future health promotion researchers, health journalists, and health policymakers. To extend our research design, we strongly encourage researchers to involve mixed-method designs, particularly combining quantitative content analysis with experimental interventions; it is more crucial to examine whether the sourcing and framing paradigms have effects on behavioral change among the newsreaders.

## Data availability statement

The raw data supporting the conclusions of this article will be made available by the authors, without undue reservation.

## Author contributions

TZ conceived the idea, conducted the literature search, developed the coding book, conducted the analysis, drafted the manuscript, and revised the final version of the manuscript. JT guided the analysis and reviewed the manuscript. XY, PC, and SK commented on the manuscript. All authors have critically reviewed, provided intellectual input to the manuscript, and approved the final version of the manuscript.

## Conflict of interest

The authors declare that the research was conducted in the absence of any commercial or financial relationships that could be construed as a potential conflict of interest.

## Publisher's note

All claims expressed in this article are solely those of the authors and do not necessarily represent those of their affiliated organizations, or those of the publisher, the editors and the reviewers. Any product that may be evaluated in this article, or claim that may be made by its manufacturer, is not guaranteed or endorsed by the publisher.
