# Supplementary material for: Sourcing and framing in cancer control continuum: A content analysis of Malaysian English and Chinese online cancer news
Source: Front Public Health. 2022 Dec 1;10:924027. doi: 10.3389/fpubh.2022.924027 (PMC9751029; doi:10.3389/fpubh.2022.924027)
Supplement: Supplementary file 1 [file Data_Sheet_1.docx]

**Table 1** | Description of coding items.

|  |  |  |  |  |  |  |  |  |
| --- | --- | --- | --- | --- | --- | --- | --- | --- |
| **News Components**  **(Coding Items)** | **Definitions** | **Origins** | ***k*** | ***The Star Online***  **n (% of total)** | ***Sin Chew Online***  **n (% of total)** | **Overall**  **n (% of total)** | $\boldsymbol{x}^{\mathbf{2}}$ | $\boldsymbol{x}^{\mathbf{2}}$  ***p*-value** |
| ***Cancer Control Continuum*** |  | Holton et al. (26) |  |  |  |  |  |  |
| Primary Cancer Prevention | The news article focuses on cancer health consultation/education and environmental risk factors control. |  | .80 | 185  (22.0%) | 234  (27.8%) | 419  (49.8%) | **19.78** | **<0.001** |
| Secondary Cancer Prevention | The news article focuses on medical interventions such as detections, screenings, and effective diagnosis approaches for one specific cancer. |  | .93 | 160  (19.0%) | 141  (16.8%) | 301  (35.8%) | 0.32 | 0.596 |
| Medical Treatment | The news article focuses on cancer medical treatment, such as an introduction for cancer medicine, a vaccine or a therapy approach which are already utilised in clinical treatment. |  | .93 | 143  (17.0%) | 90  (10.7%) | 233  (27.7%) | **11.73** | **0.001** |
| Social Support / Survivorship | The news article focuses on social support/ survivorship. For instance, either covered a campaign points to financial help, an event or an activity launched by the government, private sectors, or NGOs to attract the social attention on cancer prevention, as well as the benefits for cancer survivors. |  | .84 | 153  (18.2%) | 133  (15.8%) | 286  (34.0%) | 0.48 | 0.491 |
| Medical Research | The news article focuses on recent alternative cancer research, which may introduce a novel way to prevents cancer cause or a new medical technology relates to cancer detection, but the research findings have not been applied in the clinical treatment yet. |  | .86 | 101  (12.0%) | 73  (8.7%) | 174  (20.7%) | 3.38 | 0.066 |
| Statistical Report | The news article focuses on the statistic report on cancer incidence rate or mortality rate. |  | .84 | 48  (5.7%) | 45  (5.4%) | 93  (11.1%) | 0.002 | 0.962 |
| ***Cancer Risk Factors*** |  | Jensen et al. (3) |  |  |  |  |  |  |
| Lifestyle Risks | The news article mentions the risks related to a daily lifestyle that may cause cancer, such as alcohol consumption, smoking behaviour, physical exercise, sexual behaviour, sun exposure, obesity and mobile phone usage. |  | .93 | 100  (11.9%) | 138  (16.4%) | 238  (28.3%) | **12.84** | **<0.001** |
| Environmental/Occupational Risks | The news article mentions the risks related to the environment where the people working or living may cause cancer, such as natural resources pollutions, chemical dangerous and risky working environment. |  | 1.0 | 8  (1.0%) | 16  (1.9%) | 24  (2.9%) | 3.39 | 0.066 |
| Demographical Risks | The news article mentions the risks that related to gender, ethnicity, ageing, and socioeconomic status that may be associated with cancer occurrence. |  | .86 | 222  (26.4%) | 207  (24.6%) | 429  (51.0%) | .003 | 0.955 |
| Medical Risks | The news article mentions the risks related to certain medicine intake could increase the risk of cancer occurrence, as well as particular surgery or treatment for another disease that initially causes cancer. |  | .71 | 184  (21.9%) | 177  (21.0%) | 361  (42.9%) | 0.19 | 0.660 |
| ***News Sources*** |  | Holton et al. (26) |  |  |  |  |  |  |
| Medical Journal | The news article cited research findings from a medical journal. |  | 1.0 | 25  (3.0%) | 20  (2.4%) | 45  (5.4%) | 0.26 | 0.608 |
| Medical Institution | The news article interviewed researchers, doctors, health professionals or other staff from a medical research institution, a hospital or a university. |  | .89 | 254  (30.2%) | 208  (24.7%) | 462  (54.9%) | **4.04** | **0.045** |
| Pharmaceutical Company | The news article interviewed a pharmacist, a nutritionist, or a profitable healthcare provider from a private cancer care sector. |  | 1.0 | 8  (0.9%) | 5  (0.6%) | 13  (1.5%) | 0.50 | 0.481 |
| Government Agency | The news article cited a government report, interviewed a governmental official from the Ministry of Health and other federal or state governmental sectors. |  | .94 | 56  (6.7%) | 92  (10.9%) | 148  (17.6%) | **14.11** | <0.001 |
| NGOs | The news article interviewed the staff or cited the report from an NGO, such as the World Health Organisation (WHO) and the National Cancer Society Malaysia (NCSM). |  | .91 | 145  (17.3%) | 119  (14.1%) | 264  (31.4%) | 1.46 | 0.226 |
| Others | If the news article interviewed other individuals or cited other sources which do not belong to any of the categories above, then code the news source as others. |  | .96 | 97  (11.5%) | 79  (9.4%) | 176  (20.9%) | 0.95 | 0.329 |
| ***News Frames*** |  | Riles et al. (8) | .77 |  |  |  | **14.93** | **0.002** |
| Lifestyle Frame | The news article mainly discusses the responsibility for cancer prevention or cancer-causing in an angle of daily lifestyle choices, such as tobacco consumption and unhealthy diet intake. |  |  | 46  (5.5%) | 76  (9.0%) | 122  (14.5%) |  |  |
| Environmental Frame | The news article mainly mentions environmental issues that may cause cancer, such as environmental pollutions, nuclear radiation, and industrial/chemical pollutions. |  |  | 4  (0.5%) | 9  (1.0%) | 13  (1.5%) |  |  |
| Medical Frame | The news article mainly looks at cancer issue in a medical angle, the medical terms are cited, as well as discuses cancer prevention related to genetic or internal uncontrollable biological factors. Meanwhile, cancer care professionals or the medical journal should be cited or interviewed. It could be a coverage on the medical finding or educational information, which embark on mobilisation on cancer detection (secondary cancer prevention). |  |  | 311  (37.0%) | 265  (31.5%) | 576  (68.5%) |  |  |
| No Clear Identification |  |  |  | 75  (8.9%) | 55  (6.5%) | 130  (15.5%) |  |  |

Notes: k = Cohen’s Kappa value, $x^{2}$ = Chi square.

**Table 2 |** Logistic regression analysis: Associations between news factors and the invitation of news sources (N=841).

| **Predictors** | **News Sources**  **Odds Ratio [95% CI]** | | | | |
| --- | --- | --- | --- | --- | --- |
|  | **Medical Journal** | **Medical Institution** | **Pharmaceutical Company** | **Government Agency** | **NGOs** |
| Cancer Continuum |  |  |  |  |  |
| Primary Cancer Prevention  (ref: No) | 2.80**  [1.28-6.12] | 1.34  [0.92-1.93] | 0.22*  [0.06-0.87] | 0.74  [0.49-1.13] | 1.59*  [1.10-2.31] |
| Secondary Cancer Prevention  (ref: No) | 0.04  [0.16-.1.01] | 2.50***  [1.74-3.59] | 1.87  [0.52-6.73] | 0.73  [0.48-1.13] | 0.73  [0.50-1.08] |
| Medical Treatment  (ref: No) | 0.18*  [0.04-0.78] | 1.65**  [1.10-2.46] | 1.34  [0.40-4.49] | 0.85  [0.54-1.34] | 0.30***  [0.19-0.47] |
| Social Support / Survivorship  (ref: No) | / | 0.26***  [0.17-0.38] | / | 0.80  [0.50-1.26] | 3.18  [2.13-4.75] |
| Medical Research  (ref: No) | 6.43***  [2.85-14.54] | 11.86***  [6.52-21.56] | .67  [0.15-3.03] | 0.22***  [0.11-0.44] | 0.23***  [0.12-0.41] |
| Statistical Report  (ref: No) | 1.13  [0.40-3.24] | 0.75  [0.46-1.23] | 1.02  [0.21-4.92] | 3.55***  [2.18-5.76] | 0.66  [0.38-1.15] |
| Cancer Risk Factors |  |  |  |  |  |
| Lifestyle Risks  (ref: No) | 5.96***  [3.09-11-48] | 2.40***  [1.74-3.33] | .44  [0.10-2.03] | 0.60*  [0.39-0.92] | 0.56**  [0.39-0.79] |
| Environmental/Occupational Risks  (ref: No) | 0.48  [0.06-3.71] | 0.67  [0.29-1.57] | / | 2.88*  [1.21-6.85] | 1.12  [0.46-2.72] |
| Demographical Risks  (ref: No) | 0.57  [0.30-1.10] | 0.90  [0.68-.1.19] | .58  [0.19-1.81] | 0.89  [0.48-1.02] | 1.53**  [1.13-2.07] |
| Medical Risks  (ref: No) | 0.60  [0.30-1.22] | 1.98***  [1.48-2.64] | .81  [0.26-2.52] | 0.70  [0.48-1.02] | 0.56***  [0.30-1.19] |

Notes: *=p <.05, **=p <.01, ***=p <.001, ref: reference category.

**Table 3 |** Logistic regression analysis: Associations between news factors and the portrayal of news frames (N=841)

| **Predictors** | **News Frames**  **Odds Ratio [95% CI]**  **(Reference: No Clear Identified)** | | |
| --- | --- | --- | --- |
|  | **Lifestyle**  **Frame** | **Environmental**  **Frame** | **Medical**  **Frame** |
| ***News Source*** |  |  |  |
| Medical Journal | 17.46**  [2.23-137.00] | / | 2.56  [0.33-19.94] |
| Medical Institution | 7.63***  [3.82-15.25] | 7.25**  [1.78-29.57] | 5.31***  [3.10-9.09] |
| Pharmaceutical Company | / | / | 3.42  [0.42-27.72] |
| Government Agency | 0.81  [0.39-1.71] | 2.83  [.73-10.95] | 0.75  [0.45-1.24] |
| NGOs | 1.13  [0.60-2.12] | 1.05  [0.27-4.07] | 0.79  [0.50-1.26] |
| ***Cancer Control Continuum*** |  |  |  |
| Primary Cancer Prevention | 96.83***  [34.75-269.82] | 4.37  [0.96-.19.93] | 3.91***  [2.23-6.88] |
| Secondary Cancer Prevention | 1.33  [0.42-4.21] | 0.91  [0.08-9.74] | 17.73***  [6.70-46.69] |
| Medical Treatment | 0.99  [0.37-2.69] | / | 2.65***  [1.52-4.65] |
| Social Support / Survivorship | 0.10***  [0.04-0.22] | 0.09**  [0.02-0.48] | 0.34***  [0.19-0.61] |
| Medical Research | 8.99***  [3.12-25.90] | 3.72  [0.59-23.26] | 5.64***  [2.32-13.70] |
| Statistical Report | 1.03  [0.32-3.30] | / | 1.47  [0.61-3.54] |
| ***Cancer Risk Factors*** |  |  |  |
| Lifestyle Risks | 11.14***  [6.17-20.12] | 1.06  [0.14-8.01] | 1.80*  [1.06-3.07] |
| Environmental/Occupational Risks | / | / | / |
| Demographical Risks | 1.08  [0.64-1.80] | 0.82  [0.19-3.65] | 2.04***  [1.38-3.04] |
| Medical Risks | 1.44  [0.82-2.53] | 2.06  [0.48-8.92] | 3.10***  [2.02-4.76] |

Notes: *=p <.05, **=p <.01, ***=p <.001.
